# Continuous Visual Focal Status Epilepticus as the Primary Presentation of NMDA-R and GAD65-R Autoimmune Epilepsy

**DOI:** 10.3389/fneur.2020.598974

**Published:** 2020-11-26

**Authors:** Elma M. Paredes-Aragón, Héctor E. Valdéz-Ruvalcaba, Andrea Santos-Peyret, Marcela Cisneros-Otero, Raúl Medina-Rioja, Sandra Orozco-Suárez, Miriam M. Hernandez, Michele D. L. Breda-Yepes, Verónica Rivas-Alonso, José J. Flores-Rivera, Iris E. Martínez-Juárez

**Affiliations:** ^1^Epilepsy Clinic, National Institute of Neurology and Neurosurgery, Mexico City, Mexico; ^2^Neurology Clinic, National Institute of Neurology and Neurosurgery, Mexico City, Mexico; ^3^Neuropsychiatry Clinic, National Institute of Neurology and Neurosurgery, Mexico City, Mexico; ^4^Unit of Medical Research in Neurologic Diseases (UIMEN), Medical National Center Century XXI, Mexican Institute of Social Security, Mexico City, Mexico; ^5^Neuroimmunology Clinic, National Institute of Neurology and Neurosurgery, Mexico City, Mexico

**Keywords:** autoimmune epilepsy, epilepsy, NMDA-receptor antibodies, aura continua, epilepsia partialis continua, GAD65-receptor antibodies

## Abstract

*Epilepsia partialis continua (EPC*) has changed in its clinical and pathophysiological definition throughout time. Several etiologies have been described in addition to classic causes of EPC. The following case depicts a young woman who had a peculiar onset of epilepsy with a continuous visual aura becoming a form of chronic recurrent and non-progressive EPC. The patient was initially misdiagnosed as a non-neurological entity (assumed psychiatric in origin), but finally, an immune-mediated epilepsy was diagnosed, and EEG showed focal status epilepticus during evolution. Once the diagnosis was achieved and immune treatment was established, the patient is seizure free. Early identification of an immune basis in patients with epilepsy is important because immunotherapy can reverse the epileptogenic process and reduce the risk of chronic epilepsy. To date, this is the only case reported with EPC manifesting as a continuous visual aura associated with antiglutamic acid decarboxylase 65 (anti-GAD65) and anti-*N*-methyl-d-aspartate (anti-NMDA) antibodies.

## Introduction

Described in 1894 by the Russian neurologist, Aleksei Kozhevnikov, the term *epilepsia partialis continua* (EPC) has changed in its clinical and pathophysiological definition throughout time (1). Initially, this term was used interchangeably as an equivalent for Kozhevnikov's syndrome, mostly caused by a rare disease termed in 1958 as Rassmusen's encephalitis. This was used to describe a form of chronic focal motor EPC with ipsilateral hemiparesia/plegia and contralateral hemispheric cortical atrophy (2) that responded to immune targets of treatment and, in some cases, hemispherectomy (3). Interestingly, these terms have long evolved into a diverse clinical spectrum of the disease that involves a myriad of focal manifestations of epilepsy.

EPC was defined by the International League Against Epilepsy (ILAE) task force report on *status epilepticus* as a subsection of focal motor *status epilepticus* [focal clonic, myoclonic, or hemyclonic arrythmic movements (or jerks) that do not impair consciousness and that affect one limb or several limbs] (4). This vision of the classification of EPC has often been questioned, as there have been several reports of diverse clinical manifestations of EPC, which can vary from simple motor symptoms to complex auditory and sensorimotor auras (to name only a few) (5).

Several etiologies have been described in addition to classic causes of EPC as well (vascular, neoplastic, systemic autoimmune) (6). A systematic review (5) described additional etiologies such as tick-borne encephalitis, unspecified encephalitis, brain trauma, multiple sclerosis, systemic etiologies, mitochondrial diseases, and metabolic disorders. Interestingly, in this review, all forms of epilepsy were described, including those with non-motor symptoms. An additional description of EPC is in current use, including patient's auras. These are described as *aura continua* (AC) and include somatosensory, proprioceptive, visual, auditory, musical, olfactory, gustatory, epigastric/autonomic, anxiety, and dysmnesic episodes.

Timeframe of symptoms is of essence, and as suggested by an European epilepsy study group (7), a subclassification aids in the diagnosis and workup; this impacts the prognosis as well (8). Roughly, the classification divides EPC into four subgroups: type 1, a single episode of EPC; type 2, chronic recurrent and non-progressive EPC; type 3, chronic persistent and non-progressive; and type 4, chronic progressive EPC (5).

The following case depicts a young woman that had an epilepsy onset with AC, with chronic persistent and non-progressive EPC, misdiagnosed initially as a non-neurological entity and finally found to be an immune-mediated epilepsy.

## Case Description

A right-handed 27-year-old female, 2nd year Internal Medicine resident had no relevant family history. In her past medical history, she had an event of right optic neuritis with amaurosis that resolved without sequelae 3 years before present medical history. At that time, she was treated with intravenous steroids and was followed up by a neurologist at her hometown. No further investigations or antimyelin oligodendrocyte glycoprotein (anti-MOG) antibodies testing were performed; the optic neuritis was a single self-limited event.

She began 2 years ago with a sudden feeling of “joy” that lasted for 40s, followed by paresthesia in both inferior extremities with loss of awareness and manual automatisms with the right hand that ended with a tonic posture of the four extremities. Phenytoin (PHT) was initiated, with a diminished frequency of seizures, about once per week. One month later, during a flight, the patient experienced transient symptoms of inability to recognize faces (prosopagnosia) and incapacity to interpret words (she describes an event where she saw the word “Mexico” but read “Comiex”). Her symptoms were brief (about 1 min each) and occurred many times during that day. A diagnosis of non-motor *status epilepticus* was made with an electroencephalogram (EEG), oxcarbazepine (OXC) was initiated, PHT was discontinued, and patient became seizure free.

**Figure 1:**
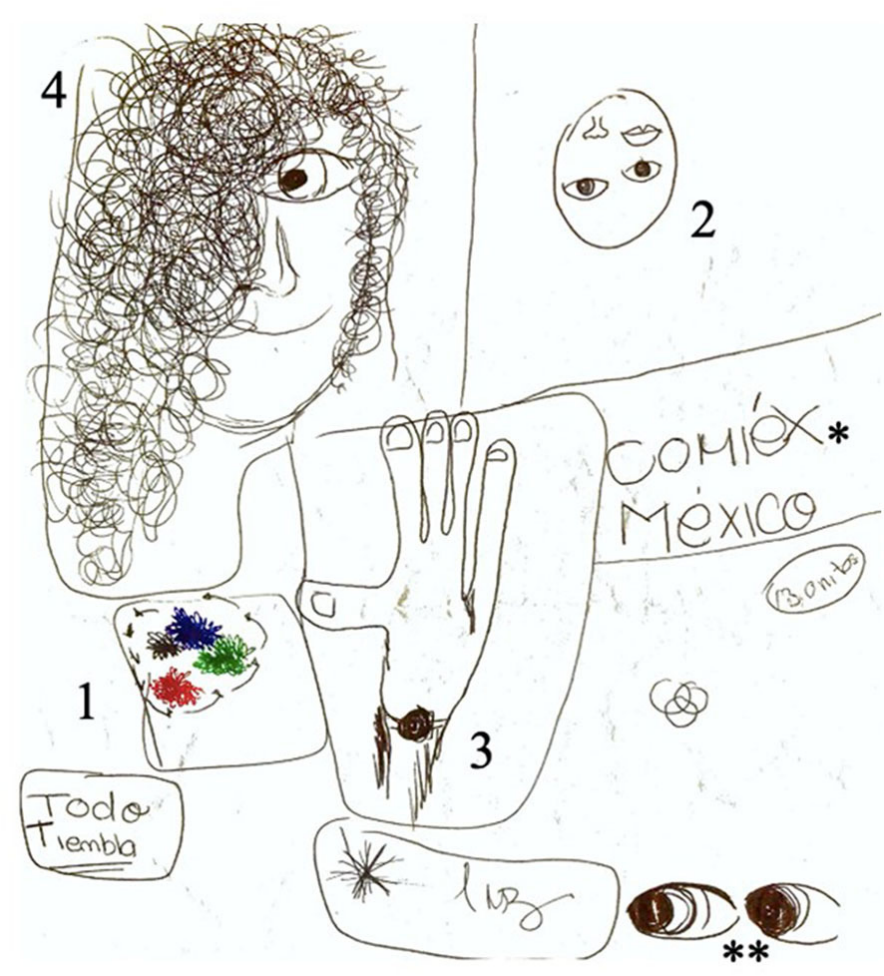
Patient's drawing depicting seizure semiology. Numbers placed in the image depict the order of symptoms: (1) flashing colors in the lower right visual field, (2) prosopagnosia, (3) visual disturbance of hand tearing in half, and (4) hair invading people's faces, fear. Isolated events that did not appear in an orderly fashion: *reading words with incapacity to interpret them; **eye version to the right.

Six months later, she developed hyponatremia due to OXC, and her antiepileptic treatment was again modified to levetiracetam (LEV). During her outpatient follow-up, she still presented with “flashing lights in colors predominantly red, blue and green in her lower right visual field,” prosopagnosia, and other visual disturbances including seeing her hand as if it were torn in half, finally followed by visual hallucinations characterized by hair invading the faces of people around her with an intense sense of fear. These always occurred in a stepwise manner, and the patient drew her seizures (Figure 1). Lacosamide (LCM) and topiramate (TPM) were added to her antiseizure (AS) treatment.

**Figure 2:**
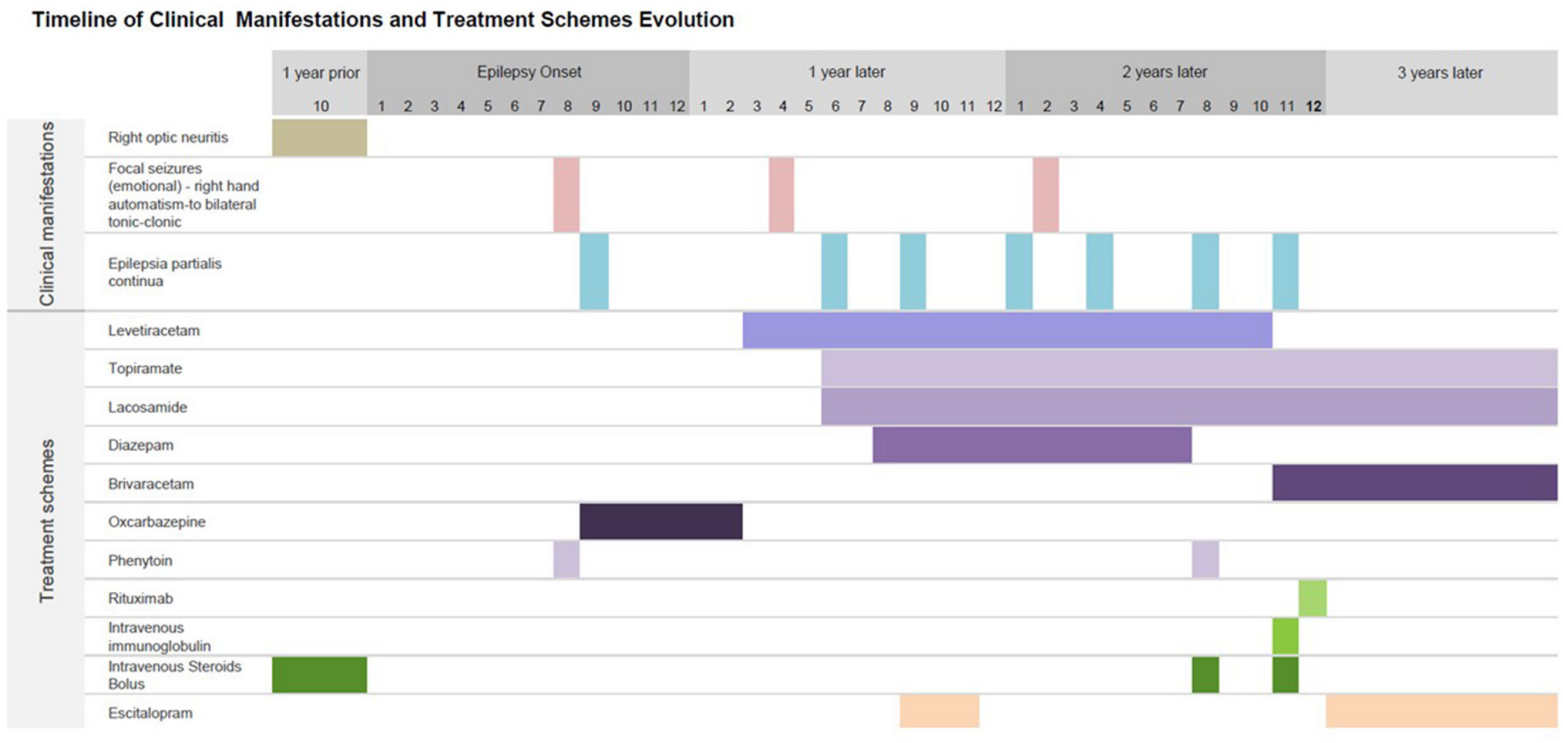
Timeline of clinical manifestations and treatment schemes. Summary of chronological findings in the patient's clinical manifestations: initial right optic neuritis 1 year prior the onset of epilepsy, followed by epilepsy onset and 1 month later appearance of chronic non-progressive recurrent epilepsia partialis continua, as described in Figure 1. These seizures could persist up to a week in duration, with fluctuating visual auras as described in detail in text. Antiseizure medications given in different time periods are also shown. Escitalopram was given at year 1 when he patient was presumed to have psychogenic non-epileptic seizures, with persistent seizures. On year 2, patient came to our Institute with epilepsia partialis continua; diagnosis workup and treatment were started. She achieved seizure freedom 3 months later.

In the course of the following year, she was admitted in second-level care centers on four occasions for treatment and observation, with recurrent EPC as described. Due to the lack of improvement in her symptoms, suspicion arose of a diagnosis of a non-neurological entity such as psychogenic non-epileptic seizures (PNES); this was assumed to be secondary to stress from workload in her residency. She was sent to a local psychiatrist, beginning treatment with fluoxetine and, due to poor response in her symptoms, was switched to escitalopram. The patient came to our third level of attention to the emergency room, as she had continued episodes as previously described for approximately 2 weeks. These episodes appeared intermittently during the day, lasting for brief seconds, but the patient constantly experienced them, which could also be present in her sleep appearing as dreams with the same manifestations (see Figure 2). Upon neurological evaluation, a cecocentral scotoma was detected (confirmed by the Neuro-ophtalmology team); the rest of her exam was normal. A conclusion of a misdiagnosis of PNES was established. IV PHT load was given (1,250 mg), and she was transferred to the Neurology ward for clinical workup.

## Diagnostic Assessment

Upon hospitalization, general laboratory tests were normal (hemoglobin, white blood cell count, liver function test, serum electrolytes, serum glucose, creatinine, and ammonium). A lumbar puncture was performed upon arrival, which showed glucose of 58 mg/dl (blood glucose was 82 mg/dl), proteins of 58 mg/dl, and three cells. Cerebrospinal fluid (CSF) was tested with Multiplex PCR FilmArray with a negative result. A brain magnetic resonance image (MRI) revealed focal restriction on diffusion weighted imaging (DWI) and fluid-attenuated inversion recovery (FLAIR) that involved the right parietotemporal region.

**Figure 3:**
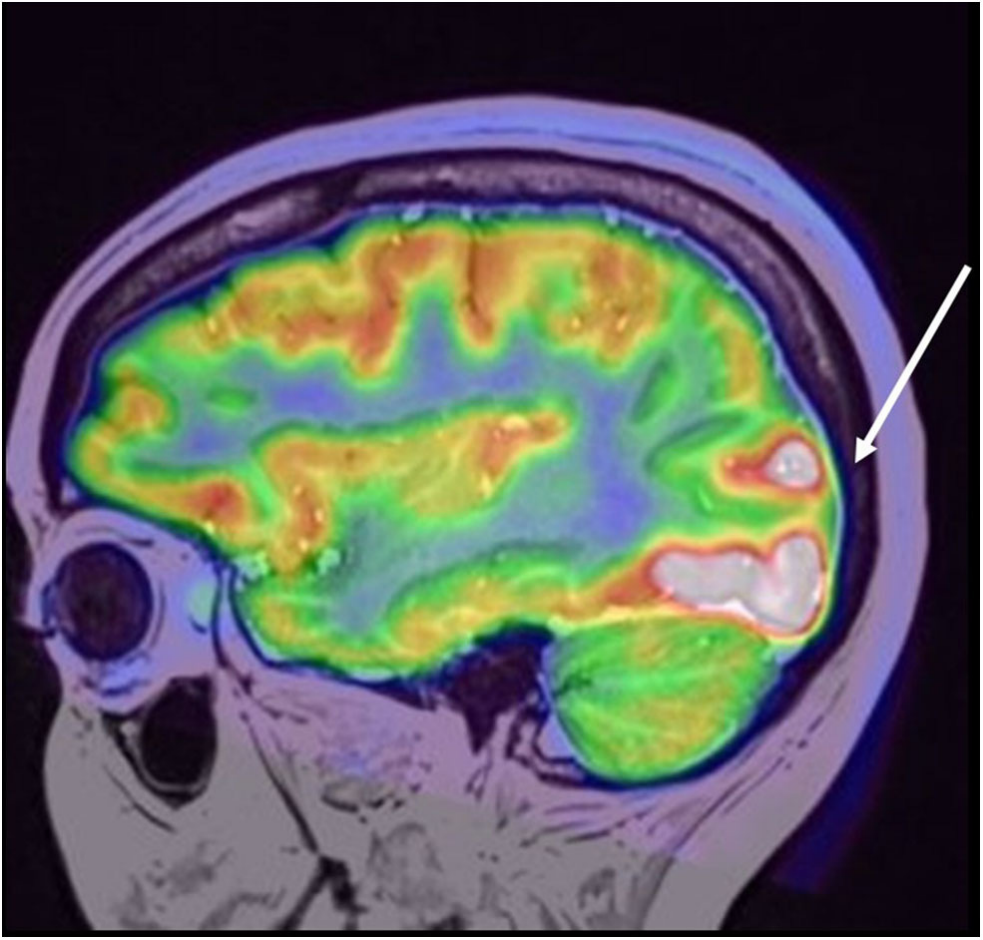
Coregistered PET/MRI study, depicting focally increased metabolic uptake in the right occipital lobe involving the lateral gyri with extension to cuneus and lingual gyri. No contralateral uptake was found or any other foci or abnormal metabolic uptake in the brain or whole body.

Contrast computed tomography of the thorax–abdomen and pelvis showed no tumor. Thyroid peroxidase (TPO) antibodies were elevated at 68.7 UI/mL (reference value, (1–16)) with normal thyroid function test. Thyroid ultrasound and transvaginal ultrasound were performed, which were also normal. Patient was evaluated by a Neuro-endocrinologist, who concluded no endocrine alteration. A full-body positron emission tomography (PET) scan showed no systemic associated tumor but a right occipital area of impressive hypermetabolism, presumed to be ictal (Figure 3).

Due to the suspicion of an autoimmune etiology, rheumatological serum analysis [complement C3 and C4, immunoglobulin G (IgG) and IgM anticardiolipin, antinuclear IgG, anticitrullinated protein, anti-Smith, IgG and IgM anti-beta-2 glycoprotein, anti-Sjögren's-syndrome-related antigen A (anti-SSA), anti-SSB, anti-DNAds, and lupus anticoagulant] was performed, and the results were also negative. CSF samples sought to inquire about antibodies against superficial neuronal antigens [antiglutamic acid decarboxylase (anti-GAD), anti-*N*-methyl-d-aspartate (anti-NMDA), anti-gamma-aminobutyric acid A (anti-GABA A) and anti-GABA B]. The identification of autoantibodies against neural antibodies by Western blot was performed by a professional team in a third-level institution specialized laboratory (view Supplementary Material). A few months later, results for surface anti-NMDA and GAD65 antibodies in CSF were positive.

## Therapeutic Interventions

Upon arrival at our Institute, autism spectrum disorder (ASD) treatment included LEV 2 g bid, TPM 300 mg bid, diazepam (DZP) 5 mg bid, and LCM 300 mg bid. Due to the diagnosis of EPC, poor response to high-dose ASD and suspicion of immunomediated mechanism prompted administration of five doses IV methylprednisolone in bolus of 1 mg each, with important clinical response. An EEG performed at that time showed right frontocentral focal slowing and epileptiform activity.

## Outcome and Follow-Up

**Figure 4:**
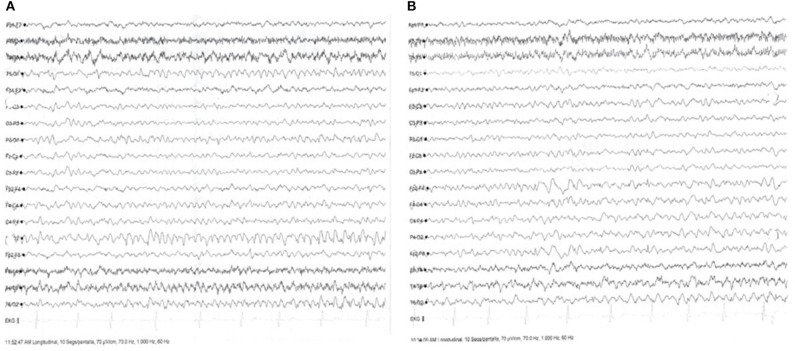
Electroencephalogram (EEG) with focal status epilepticus and after administration of antiseizure drug (AD). **(A)** Ictal surface EEG with generalized slowing predominantly in the right temporo-occipital regions, with ictal monomorphic activity in the right occipital regions, with 10 clinical events of focal motor seizures without impairment of consciousness. **(B)** Surface EEG after administration of 200 mg Brivaracetam (BRV), with resolution of ictal activity, showing focal slowing in the temporo-occipital right region.

Three months later, she continued having focal seizures with prosopagnosia in spite of good therapy adherence. Seizures worsened and appeared also at nighttime. She was readmitted to the Neurology ward, and IV methylprednisolone was readministered with no improvement; intravenous immunoglobulin (IV Ig) was given for 5 consecutive days. On the 3rd day of IV Ig treatment administration, she began with EPC, and in-hospital EEG showed continuous focal status epilepticus despite the IV Ig and the multiple ASD treatment combinations. A dose of 200 mg IV brivaracetam (BRV) was administered followed by immediate clinical and EEG resolution (Figure 4). LEV was tapered, and oral BRV was started in a dose of 100 mg bid. Treatment of IV Ig was followed by 2 g of IV rituximab. She has been seizure free since her hospital discharge.

Future estimated follow-up includes body PET scans to search for tumor association every 6 months, follow-up in outpatient clinic every 3 months for seizure control and ASD adjustment, and a lumbar puncture for antibody evaluation in 1 year. Further follow-up to search for tumor association will be up to 2 years (9).

## Discussion

To our knowledge, this is the first case of autoimmune encephalitis in adult that manifests as EPC with a continuous visual aura and in which anti-GAD and anti-NMDA antibodies coexist. We believe that the coexistence of these two antibodies could be responsible for the requirement of more than one immunotherapy and ASD in this patient. In anti-GAD autoimmune epilepsy, response to treatment is favorable in only 50% of patients in stages 1 and 2 of the disease in which there is some irreversibility of the immune process with an increase in the rate of developing chronic drug-resistant epilepsy (10). The patient's clinical presentation of simultaneous anti-GAD and anti-NMDA antibodies is unusual in clinical presentation. Although they are well-known to cause heterogeneous presentations, both have been described as part of a “classic” limbic encephalitis spectrum (mood disorders, memory impairment) as well as well-established temporal lobe epilepsy manifestations (11–14). The subacute onset of distinct manifestations of EPC, as well as the finding of mild protein increase in CSF and right neuritis optica that initially improved with immunomodulation in a previously healthy patient, suggests a role of autoimmunity involving these antibodies with a possibly broader clinical spectrum. Moreover, this is the first case reported in the literature with EPC presenting as aura continua in an adult patient with autoimmune encephalitis.

Focal seizures are a common symptom in autoimmune encephalitis (AE). A predisposition to cause seizures is due to a mechanism that boosts the immune response either mediated by both antibodies (surface antigens) or mediated by cytotoxic *T* cells (intracellular antigens) (11). Additional studies describe autoimmune encephalitis as the cause of 20% of adult epilepsy of unknown etiology (15, 16). In general, seizures are usually the first clinical manifestation, as with our patient. They can become the predominant feature or even the only clinical manifestation of the disease (11, 15). In an Iranian series of 33 adult patients with focal epilepsy with and without mesial temporal sclerosis, the prevalence of autoimmune epilepsy was 50%. The most frequent antibodies were anti-gamma-aminobutyric acid receptor (GABA-R) in 33% of the patients followed by anti-NMDA receptor (anti-NMDA-R) in 6% and anti-GAD in 3%, in agreement with other published series. None of these patients presented with status epilepticus (17). There have been reports of EPC in patients with NMDA and GAD65 autoantibody encephalitis involving focal motor status (14, 18). The initial manifestation of right neuritis optica that responded to steroid treatment raises the possibility of the previously described overlap between demyelinating diseases and NMDA-R autoantibodies (acute disseminated encephalomyelitis, myelitis, neuromyelitis optica). Recently, Taraschenko et al. narrated a case of a patient with classic limbic encephalitis associated with anti-MOG antibodies showing involvement of both optic nerves in brain MRI. Although imaging and CSF findings were not compatible or suggestive of any demyelinating disorder, the question of overlap of these entities as a catalyst for initial neuritis optica is certainly feasible (19).

It is reported that the coexistence of multiple antibodies in autoimmune encephalitis increases the risk of underlying malignancy and may be predictive of a specific type of malignancy according to the antibody (20). The probability of malignancy with anti-GAD65 antibodies increases if the presentation meets the classic presentation of a paraneoplastic syndrome or if coexisting antibodies against the neuronal cell surface are present (21); however, our patient has not showed so far any associated malignancy in her screening. In anti-GAD encephalitis, responder rates to corticosteroid and IV Ig therapy are generally lower than other neural antibody-mediated syndromes. Response rates depended on the time of initiation of the immunotherapy, with a poor prognosis and a greater risk of chronic epilepsy in patients with a delay in treatment initiation (10, 22–24). The presence of antibodies against glutamic acid decarboxylase (GAD-abs) is related to a variety of clinical syndromes such as stiff-person syndrome (SPS), limbic encephalitis (LE), epilepsy, cerebellar ataxia, diabetes mellitus type I, and other autoimmune endocrine disorders (25). The positivity of these antibodies is strongly associated with thyroid disease. TPO titers in our patient were elevated, but no associated thyroid disease was found by imaging and even with expert Neuro-endocrine evaluation. An explanation of the heterogeneity of the clinical presentation is the differences in tissue distribution and the specificity of the epitope of anti-GAD reactivity in different conditions. It has been observed that the serum of patients with epilepsy shows more reactivity against the carboxy or C terminal domain (21, 25–27). We believe that these findings in our patient were related to anti-GAD reactivity.

Another clinical spectrum that has been associated with elevated TPO titers is steroid responsive autoimmune encephalitis (formerly known as Hashimoto encephalitis). Due to its “low prevalence” and varied clinical presentation, no current recognized diagnostic criteria exist. However, it is generally accepted that the diagnosis must include encephalopathy, cognitive dysfunction, and psychiatric features, along with high TPO titers, response to treatment with steroids, and exclusion of another neurological disease (28). In a study by Mattozzi et al., 24 patients with clinical criteria for Hashimoto encephalitis also fulfilled criteria for other autoimmune-mediated syndromes, such as possible autoimmune encephalitis and limbic encephalitis. Steroid response was poor (31%), the TPO titers were considered not disease specific. It was concluded that current criteria for Hashimoto encephalitis is unable to discriminate between the disease itself and other conditions (29). Interestingly, high TPO titers have been linked to epilepsia partialis continua (with motor manifestations). The first case was described by Zeynep Aydin-Ozemir at al. A young female patient developed focal motor EPC, drug resistant with extensive bifrontal and Rolandic gyrus involvement on MRI brain, with a normal lumbar puncture. The patient was found to have elevated TPO values (with seric euthyroid results) and radionuclide scan with an elevated and homogeneous uptake of the thyroid gland (30). Our patient had elevated TPO titers; the clinical presentation and no abnormal structural finding in the thyroid gland upon investigation as well as other CSF antibodies made the diagnosis quite unlikely.

The response to treatment is 70–80% effective in patients with anti-NMDA encephalitis with a risk of conversion to chronic epilepsy in <5%. Regarding anti-GAD65-mediated autoimmune epilepsy, the percentage of seizure persistence associated with limbic encephalitis ranges from 5 to 12.5% in adult epilepsy and over 6% in childhood epilepsy (22). Specifically, in an Italian retrospective cohort by Casciato et al., 33 patients with limbic encephalitis (LE) were studied; 100% of patients were found to develop seizures initially, and upon 12-month follow-up, 13/33 (39.3%) developed epilepsy. Interestingly, the study found 12/33 (36%) cases with positive antibodies: seven patients with anti-VGKC (LGI-1 in 3 and CASPR-2 in 4), two with anti-NMDAR antibodies, and finally, one patient with Abs anti-SOX-1, one with Ri/Hu, and one with GAD65, illustrating the occurrence of these findings in many autoimmune-mediated epilepsy presentations. Cases that developed chronic epilepsy (39.3%) were found to be associated statistically with predisposing factors: diagnostic delay, low seizure frequency at onset, absence of amnestic syndrome, and absence of EEG discharges (31).

Our patient had many peculiarities: seizure characteristics were unusual (aura continua with visual status), misdiagnosis that delayed seizure treatment optimization, and a mixed presentation of GAD65 and NMDA-R antibody presentation. This conveyed challenges in treatment for the patient, who received an optimal combination of ASD as well as immunomediated treatment. In spite of the optimal treatment, she presented with refractory status epilepticus as visual EPC during her 3rd day of IV Ig. The efficacy of BRV has been well-established in status epilepticus, as an effective and tolerable ASD, which can be administered intravenously (32, 33). The patient was already with LEV treatment, but BRV was more effective in achieving seizure remission in this case. The reason for this is not certain, but some hypotheses can be made. The effectiveness of BRV over LEV lies on its affinity to SV2A receptors estimated to be 15–30 times more potent than LEV and with faster brain permeability, which is especially important in the urgent scenario of EPC (34). Switching an ASD treatment from LEV to BRV is certainly preferred in patients with autoimmune encephalitis, as many may have neuropsychiatric symptoms as well (35).

The main goal of this case report is to convey the potential harm when there is delay in the diagnosis of a case of autoimmune epilepsy and the misdiagnosis of some of the seizures particularly in this patient that had visual EPC as non-neurological events. The early identification of an immune basis in patients with epilepsy is fundamental because immunotherapy can reverse the epileptogenic process and reduce the risk of chronic and refractory epilepsy.

## Data Availability Statement

The raw data are not available due to patient's confidentiality.

## Ethics Statement

Written informed consent was obtained from the individual(s) for the publication of any potentially identifiable images or data included in this article.

## Author Contributions

EP-A was an epilepsy fellow that began the initial workup, treatment, follow-up of the patient, and wrote and edited the article. HV-R was involved in planning the article in redaction and discussion. MC-O was in charge of image edition. RM-R wrote the first draft of the case report. SO-S, MH, MB-Y, VR-A, JF-R, and AS-P were involved in writing and in editing. NEKR supervised image editing, CCA performed image upload. IM-J was the supervisor for this article and has since then treated the patient. All authors contributed to the article and approved the submitted version.

## Conflict of Interest

The authors declare that the research was conducted in the absence of any commercial or financial relationships that could be construed as a potential conflict of interest.

## References

[1] Bancaud J Bonis A Trottier S Talairach J Dulac O. Continuous partial epilepsy: syndrome and disease. Rev Neurol (Paris). (1982) 138:803–14. Available online at: http://www.ncbi.nlm.nih.gov/pubmed/6820177 (accessed February 5, 2020). 6820177

[2] Vein AA Van Emde Boas W. Kozhevnikov epilepsy: the disease and its eponym. Epilepsia. (2011) 52:212–8. 10.1111/j.1528-1167.2010.02900.x 21204824

[3] Varadkar S Bien CG Kruse CA Jensen FE Bauer J Pardo CA. Rasmussen's encephalitis: clinical features, pathobiology, and treatment advances. Lancet Neurol. (2014) 13:195–205. 10.1016/S1474-4422(13)70260-6 24457189 PMC4005780

[4] Trinka E Cock H Hesdorffer D Rossetti AO Scheffer IE Shinnar S. A definition and classification of status epilepticus - report of the ILAE task force on classification of status epilepticus. Epilepsia. (2015) 56:1515–23. 10.1111/epi.13121 26336950

[5] Mameniškiene R Wolf P. Epilepsia partialis continua: a review. Seizure. (2017) 44:74–80. 10.1016/j.seizure.2016.10.010 28029552

[6] Bien CG Elger CE. Epilepsia partialis continua: semiology and differential diagnoses. Epileptic Disord. (2008) 10:3–7. 10.1684/epd.2008.0161 18367424

[7] Mameniskiene R Bast T Bentes C Canevini MP Dimova P Granata T. Clinical course and variability of non-Rasmussen, nonstroke motor and sensory epilepsia partialis continua: a European survey and analysis of 65 cases. Epilepsia. (2011) 52:1168–76. 10.1111/j.1528-1167.2010.02974.x 21320117

[8] Gutiérrez-Viedma Romeral-Jiménez M Serrano-García I Parejo-Carbonell B Cuadrado-Pérez ML Sanz-Graciani I García-Morales I. The importance of timing in epilepsia partialis continua. Neurologia. (2019). [Epub ahead of print]. 10.1016/j.nrl.2019.03.004. 35595402

[9] Soares EMV Gomes Kauark RB Guimarães Rocha MS Dozzi Brucki SM Encefalite anti-NMDA-R: Seguimento de 24 meses. Dement e Neuropsychol. (2013) 7:304–7. 10.1590/S1980-57642013DN70300012 PMC5619203 29213855

[10] Mäkelä KM Hietaharju A Brander A Peltola J. Clinical management of epilepsy with glutamic acid decarboxylase antibody positivity: the interplay between immunotherapy and anti-epileptic drugs. Front Neurol. (2018) 9:579. 10.3389/fneur.2018.00579 30057567 PMC6053535

[11] Geis C Planagumà J Carreño M Graus F Dalmau J. Autoimmune seizures and epilepsy. J Clin Invest. (2019) 129:926–40. 10.1172/JCI125178 30714986 PMC6391086

[12] Dalmau J Graus F. Antibody-mediated encephalitis. N Engl J Med. (2018) 378:840–51. 10.1056/NEJMra1708712 29490181

[13] Dalmau J Gleichman AJ Hughes EG Rossi JE Peng X Lai M. Anti-NMDA-receptor encephalitis: case series and analysis of the effects of antibodies. Lancet Neurol. (2008) 7:1091–8. 10.1016/S1474-4422(08)70224-2 18851928 PMC2607118

[14] Petit-Pedrol M Armangue T Peng X Bataller L Cellucci T Davis R. Encephalitis with refractory seizures, status epilepticus, and antibodies to the GABA A receptor: a case series, characterisation of the antigen, and analysis of the effects of antibodies. Lancet Neurol. (2014) 13:276–86. 10.1016/S1474-4422(13)70299-0 24462240 PMC4838043

[15] Spatola M Dalmau J. Seizures and risk of epilepsy in autoimmune and other inflammatory encephalitis. Curr Opin Neurol. (2017) 30:345–53. 10.1097/WCO.0000000000000449 28234800 PMC5831325

[16] Dubey D Alqallaf A Hays R Freeman M Chen K Ding K. Neurological autoantibody prevalence in epilepsy of unknown etiology. JAMA Neurol. (2017) 74:397–402. 10.1001/jamaneurol.2016.5429 28166327

[17] Ansari B Etemadifar M Najafi M Nasri M Meamar R Neuronal autoantibodies in focal epilepsy with or without mesial temporal sclerosis. Iran J Neurol. (2019) 18:13–8. 10.18502/ijnl.v18i1.941 31316731 PMC6626608

[18] Olson JA Olson DM Sandborg C Alexander S Buckingham B. Type 1 diabetes mellitus and epilepsia partialis continua in a 6-year-old boy with elevated anti-GAD65 antibodies. Pediatrics. (2002) 109:E50. 10.1542/peds.109.3.e50 11875178

[19] Taraschenko O Zabad R. Overlapping demyelinating syndrome and anti-N-methyl-D-aspartate receptor encephalitis with seizures. Epilepsy Behav Reports. (2019) 12:100338. 10.1016/j.ebr.2019.100338 31737864 PMC6849071

[20] Kammeyer R Piquet al. Multiple co-existing antibodies in autoimmune encephalitis: a case and review of the literature. J Neuroimmunol. (2019) 337:577084. 10.1016/j.jneuroim.2019.577084 31655424

[21] Ariño H Höftberger R Gresa-Arribas N Martínez-Hernández E Armangue T Kruer MC. Paraneoplastic neurological syndromes and glutamic acid decarboxylase antibodies. JAMA Neurol. (2015) 72:874–81. 10.1001/jamaneurol.2015.0749 26099072 PMC4838033

[22] Daif A Lukas RV Issa NP Javed A VanHaerents S Reder AT. Antiglutamic acid decarboxylase 65 (GAD65) antibody-associated epilepsy. Epilepsy Behav. (2018) 80:331–6. 10.1016/j.yebeh.2018.01.021 29433947

[23] Lilleker JB Biswas V Mohanraj R. Glutamic acid decarboxylase (GAD) antibodies in epilepsy: diagnostic yield and therapeutic implications. Seizure. (2014) 23:598–602. 10.1016/j.seizure.2014.04.009 24836709

[24] Quek AML Britton JW McKeon A So E Lennon VA Shin C. Autoimmune epilepsy: clinical characteristics and response to immunotherapy. Arch Neurol. (2012) 69:582–93. 10.1001/archneurol.2011.2985 22451162 PMC3601373

[25] Saiz A Blanco Y Sabater L González F Bataller L Casamitjana R. Spectrum of neurological syndromes associated with glutamic acid decarboxylase antibodies: diagnostic clues for this association. Brain. (2008) 131:2553–63. 10.1093/brain/awn183 18687732

[26] Gresa-Arribas N Ariño H Martínez-Hernández E Petit-Pedrol M Sabater L Saiz A. Antibodies to inhibitory synaptic proteins in neurological syndromes associated with glutamic acid decarboxylase autoimmunity. PLoS ONE. (2015) 10:e0121364. 10.1371/journal.pone.0121364 25774787 PMC4361655

[27] Liimatainen S Honnorat J Pittock SJ McKeon A Manto M Radtke JR. GAD65 autoantibody characteristics in patients with co-occurring type 1 diabetes and epilepsy may help identify underlying epilepsy etiologies. Orphanet J Rare Dis. (2018) 13:55. 10.1186/s13023-018-0787-5 29636076 PMC5892043

[28] Zhou JY Xu B Lopes J Blamoun J Li L. Hashimoto encephalopathy: literature review. Acta Neurol Scand. (2017) 135:285–90. 10.1111/ane.12618 27324276

[29] Mattozzi S Sabater L Escudero D Ariño H Armangue T Simabukuro M. Hashimoto encephalopathy in the 21st century. Neurology. (2020) 94:e217–24. 10.1212/WNL.0000000000008785 31882532

[30] Aydin-Özemir Z Tüzün E Baykan B Akman-Demir G Özbey N Gürses C. Autoimmune thyroid encephalopathy presenting with epilepsia partialis continua. Clin EEG Neurosci. (2006) 37:204–9. 10.1177/155005940603700308 16929705

[31] Casciato S Morano A Fattouch J Fanella M Avorio F Albini M. Factors underlying the development of chronic temporal lobe epilepsy in autoimmune encephalitis. J Neurol Sci. (2019) 396:102–7. 10.1016/j.jns.2018.10.026 30447604

[32] Farrokh S Bon J Erdman M Tesoro E. Use of newer anticonvulsants for the treatment of status epilepticus. Pharmacother J Hum Pharmacol Drug Ther. (2019) 39:297–316. 10.1002/phar.2229 30723940

[33] Brigo F Lattanzi S Nardone R Trinka E. Intravenous brivaracetam in the treatment of status epilepticus: a systematic review. CNS Drugs. (2019) 33:771–81. 10.1007/s40263-019-00652-0 31342405

[34] Feyissa AM. Brivaracetam in the treatment of epilepsy: a review of clinical trial data. Neuropsychiatr Dis Treat. (2019) 15:2587–600. 10.2147/NDT.S143548 31571877 PMC6750854

[35] Kalss G Rohracher A Leitinger M Pilz G Novak HF Neuray C. Intravenous brivaracetam in status epilepticus: a retrospective single-center study. Epilepsia. (2018) 59:228–33. 10.1111/epi.14486 30043427

