# Supplementary material for: Continuous Visual Focal Status Epilepticus as the Primary Presentation of NMDA-R and GAD65-R Autoimmune Epilepsy
Source: Front Neurol. 2020 Nov 26;11:598974. doi: 10.3389/fneur.2020.598974 (PMC7726346; doi:10.3389/fneur.2020.598974)
Supplement: Supplementary file 1 [file Data_Sheet_1.docx]

Supplementary Material

# Supplementary Data

Methods of Western Blot identification of Surface Antibodies

An extraction of BALCE rat brain protein utilizing a tampon with lysis and protease inhibitors (Tris-HCl 50 mM, pH 8.0, sodium chloride 150 mM, Igepal CA-630 [NP-40] in 1.0%, 0.5%, sodium desoxicolate and 0.1 % of docecyl sodium sulfate, Sigma Aldrich /protease inhibitors, ROCHE), was centrifuged to 18,000g for 15 minutes. The pill will be soluble for sonification in Triton X-100 to 2%, buffer trisbarbital 70mM, pH 8.6, NaN3 15 mM, phenyl-methyl-sulphonyl 0.1 mM (PMSF) and 100 U/ml of apoprotinine. Following this step, the sample was cetrifuged for 15 minutes at a rate of 18,000 g/15 min. The resting sample was placed in dodecil-sodium sulfate (SDS) (Tris/Hcl buffer, pH 7.2, 2.3% w/v SDS, 3% v/v glicerol) that must contain 5% β-mercaptanol. It was held in a polyacrilamide gel at a concentración de10%. Proteins were charged in a concentration of 70µl protein simple in 50µl of distilled wáter and 30µl of LB, obtaining a final concentration of 150µl per aliquot. Of this sample, 30µl of charge simple was collected, and finally a gel electrophoresis in 10% poliacrilamide to 85volt for 2:30h. These were transported in(Tris-HCl 50 mM, pH 8.0, with sodium chloride150 mM, Igepal CA-630 [NP-40] to 1.0%, 0.5%, sodium desoxicolate and 0.1 % sodium dodecilsulfate, Sigma Aldrich / protease inhibitors, ROCHE), will be centrifuged to 18,000g for 15 minutes. The pill was soluble for sonification in tritonX-100 in 2%, a buffer with trisbarbital 70 mM, pH 8.6, NaN3 15 mM, florurum of phenyl-methyl-sulphonyl 0.1mM (PMSF) and 100U/ml of aprotinine, will be centrifuged for 15 minutes to 18,000 g/15 min. Phenyl-methyl-sulphonyl fluoride 0.1 mM (PMSF) and 100 U/ml of aprotinine, followed by a 15 minute centrifuge at 18,000 g/ per 15 minutes. The supernatant will be plaed in dodecil-sodium-sulfate (SDS) (Tris/Hcl buffer, pH 7.2, 2.3% w/v SDS, 3% v/v glicerol) which must contain 5% β-mercapthanol and will be run with a gel of polyachrilamide at a concentration of10% Proteins will be charged at a concentrarion of 70µl of 50µl protein sample of distilled water and 30µl of LB, obtaining a final concentration of 150µl per aliquot, taking a sample of only 30µl per charge, with a gel electrophoresis of 10% polyacrylamide in 85 volts per 2:30 hurs. They will be transferred to a Difluorate Polivinyl membrane(PVDF) by a camera of semi-humid transference, applying 20 volts for 1 hour. Non specific unions will be blocked inclubating with Svelty milk 0.5% diluted in a Buffer PBS-Twen (Tris (Tris 50 mM, pH 10.2, NaCl 350 mM, PSMF 0.2mM) during 1 hour. It will be incubated for 24 hours with CSF and plasma1:1000. Three lavages will be performed with PBS-Twen each for 5 minutes and a secondary IgG antibody will be applied marked with peroxidase and will be detected with Millpore Crescent chemoluminiscence . Proteins will be visualized using a Fusion-Fx Vilbert Loumart photodocumenter. Molecular weight are calculated, recognizing circulating IgG antibodies in CSF and/or plasma.

**Do Blot technique for the identification of circulating antigens in CSF and plasma**

Over a membrane of nitrocellulose in the Do blot equipment by Biorad, 10 and 5 ul of CSF and plasma adhere to membranes by vacuum and after this the membrane dries at room temperature. It is blocked by saline solution ofphosphates (PBS)- milk 5%. After three washing cycles with PBS-Tween 20 (PBS-T) during 5 minutes, these are incubated with each primal antibody to study NMDAR1 and GAD65/67 (Santa Cruz Labs) at a dilution of 1/500 inn PBS-milk 1%. After a new washing stafe (3 times), it will be incubated with IgG anti-rat cnjugated with peroxidase, diluted in 1/500 with PBS milk 1%. The reaction is stopped with a stage of water lavage. All the incubation stages are performed with moderate agitation. As a positive control, proteic rat tissue is extracted and will be detected with a Crescent of Millipore chemomoluminiscence reactant. Proteins will be visualized with a Fusion-Fx Vilbert Loumart photodocumeter.

**TBA Assays**

With Tissue brain adhesion (TBA), sagital brain sections of Balce mice with 30um are iincubated with patient CSF or serum of patients at a dilution of 1:200 followed by three PBS lavages that will be incubated with a secondary IgG anti-human antibody marked with IFTC, followed by the incubation of a primary antibody against the protein suspected (NMDAR1, GAD65/67; Santa Cruz, lab) for 12 hours followed by three lavages that will be incubated with a secondary antibody agents the primary species marked with ALEXA -546, are mounted with Vectashie and are observed in a microscope with focal lens. The sited of recognition for NMDAR1 and GAD65/67 and must be observed in hippocampus and cerebellum.

## Supplementary Image

**
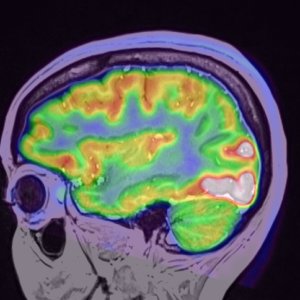
**

Supplementary Image 1. Co registered PET/MRI depicting increased focal metabolic uptake in the occipital lobe (lateral gyri) ando extension to cuneus and lingual gyri
